# Muscle-specific deletion of SOCS3 increases the early inflammatory response but does not affect regeneration after myotoxic injury

**DOI:** 10.1186/s13395-016-0108-4

**Published:** 2016-10-24

**Authors:** Kristy Swiderski, Savant S. Thakur, Timur Naim, Jennifer Trieu, Annabel Chee, David I. Stapleton, René Koopman, Gordon S. Lynch

**Affiliations:** Basic and Clinical Myology Laboratory, Department of Physiology, The University of Melbourne, Melbourne, 3010 Australia

**Keywords:** SOCS3, Muscle, Regeneration, Inflammation, MCK

## Abstract

**Background:**

Muscles of old animals are injured more easily and regenerate poorly, attributed in part to increased levels of circulating pro-inflammatory cytokines. The Janus kinase/signal transducers and activators of transcription (JAK/STAT) signaling cascade is a key mediator of inflammatory cytokine action, and signaling via this pathway is increased in muscles with aging. As a negative regulator of JAK/STAT signaling, a key mediator of myogenic proliferation and differentiation, altered expression of suppressor of cytokine signaling (SOCS3) is likely to have important consequences for muscle regeneration. To model this scenario, we investigated the effect of SOCS3 deletion within mature muscle fibers on injury and repair. We tested the hypothesis that reduced SOCS3 function would alter the inflammatory response and impair muscle regeneration after myotoxic injury.

**Methods:**

Mice with a specific deletion of SOCS3 within mature skeletal muscle fibers were used to assess the effect of SOCS3 deletion on muscle injury and repair. Twelve-week-old or 24-month-old SOCS3 muscle-specific knockout (SOCS3 MKO) mice and littermate controls were either left uninjured or injured with a single injection of notexin (10 μg/ml) into the right tibialis anterior (TA) muscle. At 1, 2, 3, 5, 7, or 14 days post-injury, the right TA muscle was excised and subjected to histological, western immunoblotting, and gene expression analyses. Force production and fatigue were assessed in uninjured muscles and at 7 days post-notexin injury.

**Results:**

In uninjured muscles, SOCS3 deletion decreased force production during fatigue but had no effect on the gross or histological appearance of the TA muscles. After notexin injury, deletion of SOCS3 increased STAT3 phosphorylation at day 1 and increased the mRNA expression of the inflammatory cytokine *TNF-α*, and the inflammatory cell markers *F4/80* and *CD68* at day 2. Gene expression analysis of the regeneration markers *Pax7*, *MyoD*, and *Myogenin* indicated SOCS3 deletion had no effect on the progression of muscle repair after notexin injury. Inflammation and regeneration were also unchanged in the muscles of 24-month-old SOCS3 MKO mice compared with control.

**Conclusions:**

Loss of SOCS3 expression in mature muscle fibers increased the inflammatory response to myotoxic injury but did not impair muscle regeneration in either adult or old mice. Therefore, reduced SOCS3 expression in muscle fibers is unlikely to underlie impaired muscle regeneration. Further investigation into the role of SOCS3 in other cell types involved in muscle repair is warranted.

**Electronic supplementary material:**

The online version of this article (doi:10.1186/s13395-016-0108-4) contains supplementary material, which is available to authorized users.

## Background

Muscles of old animals are more susceptible to injury and regenerate poorly. Cycles of repeated damage and incomplete repair contributes to muscle atrophy and weakness with age [1–5]. A well-regulated inflammatory response is critical for the initiation of muscle repair through muscle stem cell activation and necessary for myogenic differentiation [6–11]. In contrast, chronic low-grade systemic inflammation is thought to interfere with effective regeneration in older individuals. Although increased levels of circulating pro-inflammatory cytokines, such as interleukin −6 (IL-6), interferon-γ (IFN-γ), and tumor necrosis factor-α (TNF-α) are associated with muscle wasting and are increased in aged muscle [12], the signaling mechanisms controlling degeneration, regeneration, and inflammation are not well understood.

Inflammatory cytokines exert many effects via activation of the Janus kinase/Signal transducers and activators of transcription (JAK/STAT) signaling pathway [13]. The suppressors of cytokine signaling (SOCS) protein family consisting of eight members including cytokine-induced STAT inhibitor (CIS) and SOCS1-7 are key negative regulators of JAK/STAT signaling. SOCS3 is one of the best characterized SOCS proteins and an important regulator of JAK1/STAT3 signaling and inflammation in many cell types via direct interactions with both the gp130 receptor and JAK1 [14–17]. Properly regulated JAK/STAT signaling is required for progression through myogenic differentiation and critical for muscle regeneration [18].

In the absence of inflammation, SOCS3 is expressed at very low levels but is thought to play a role in various muscle resident cells including hematopoietic cells, muscle stem cells, and mature muscle fibers [17, 19–23]. In vitro adenoviral overexpression of SOCS3 in human myotubes indicated a role for SOCS3 in directing the expression of genes regulating myogenic differentiation, myotube maturation, and cell survival [24], demonstrating a potential role for SOCS3 in regulating myogenesis. Transgenic muscle-specific overexpression of SOCS3, driven by the myosin light chain (MLC) promoter, impairs muscle morphology and ambulation, associated with disrupted calcineurin signaling and defects in sarcoplasmic reticulum and mitochondrial function [25]. In another study, transgenic overexpression of SOCS3 driven by the muscle creatine kinase (MCK) promoter impaired glucose and insulin tolerance as a result of suppressed leptin-induced activation of the AMP-regulated protein kinase (AMPK) [23]. In contrast, mice with MCK-Cre-mediated SOCS3 deletion had normal muscle development and functional performance and, consistent with a role of SOCS3 in inhibition of insulin signaling, had improved insulin sensitivity and glucose homeostasis [26].

As JAK/STAT signaling is implicated in the regulation of inflammation, anabolic signaling, and myogenic differentiation, altered regulation of this signaling is likely to have important consequences for muscle health and effective regeneration. Increased STAT3 phosphorylation and SOCS3 messenger RNA (mRNA) expression has been reported in skeletal muscles from old compared with young rats [27]. SOCS3 gene and protein levels were increased in human skeletal muscle biopsies obtained from older participants (70 ± 0.3 years) compared to healthy young adults (20 ± 0.2 years) at rest [28]. However, in a different study, STAT3 activation was increased and SOCS3 protein decreased in skeletal muscle biopsies obtained from older adults compared with younger adults 2 h after a bout of resistance exercise [29]. Interestingly, mRNA levels of SOCS3 were higher in skeletal muscle biopsies obtained from older adults compared with young adults at this time, indicating either an issue with SOCS3 protein translation or a delay in the signaling pathway in muscles from older adults [29].

Studies testing the hypothesis that altered SOCS3 expression and inflammatory JAK/STAT signaling contributes to impaired muscle regeneration have produced conflicting results, but a common theme is an age-related increase in STAT3 signaling in skeletal muscle which suggests impaired negative regulation of JAK/STAT signaling by SOCS3 [27, 29]. Reduced SOCS3 function could contribute to impaired muscle regeneration and chronic inflammation with aging. To model this scenario, we investigated the effect of SOCS3 deletion, specifically within mature muscle fibers, on injury and repair. Since SOCS3 is expressed in multiple cell types involved in the repair of muscle after injury (including muscle fibers, stem cells, and inflammatory cells), it is important to determine in which cell population reduced SOCS3 expression contributes to impaired muscle regeneration. We tested the hypothesis that reduced SOCS3 function would alter the inflammatory response and impair muscle regeneration after myotoxic injury.

## Methods

### Cell culture

C2C12 cells (obtained from ATCC, Manassas, VA, USA) were maintained as proliferating myoblasts in culture at 37 °C + 5 % CO_2_ in DMEM/10 % FBS/1 % l-glutamine and sub-cultured at 60–70 % confluency. For treatment of proliferating myoblasts, C2C12 cells were seeded into six-well tissue culture plates and grown to 50–70 % confluency over 48 h. Cells were either left unstimulated (0) or stimulated with 100 ng/mL recombinant murine (rm) IL-6 (kindly provided by Prof. Warren Alexander, The Walter and Eliza Hall Institute, Melbourne, Australia), 100 ng/mL rm IFN-γ (R&D Systems Inc., MN, USA), or 10 ng/mL rm TNF-α (R&D Systems Inc.) in DMEM/10 % FBS/1 % l-glutamine for 0.25, 0.5, 1, 2, or 4 h at 37 °C ± 5 % CO_2_. For treatment of differentiated myotubes, C2C12 cells were seeded into six-well tissue culture plates and grown to 100 % confluency over 48 h at 37 °C ± 5 % CO_2_ in DMEM/10 % FBS/1 % l-glutamine. Once confluent, media were replaced with DMEM/2 % HS/1 % l-glutamine to induce myotube differentiation. Media were changed every 48 h. On day 5 of differentiation, cells were either left unstimulated (0) or stimulated with 100 ng/mL rm IL-6, 100 ng/mL rm IFN-γ, or 10 ng/mL rm TNF-α in DMEM/2 % HS/1 % l-glutamine for 0.25, 0.5, 1, 2, or 4 h at 37 °C ± 5 % CO_2_. Fresh cell culture media was not added on the day of stimulation to avoid activation of signaling pathways via addition of fresh serum.

### Animals

SOCS3^fl/fl^ MCK-Cre mice were obtained from Prof. Gregory Steinberg (St Vincent’s Institute, Melbourne, Australia) and bred and maintained in the Biological Research Facility (BRF) at The University of Melbourne. Breeding was performed to generate animals heterozygous for Cre-recombinase expression (SOCS3^fl/fl^ MCK-Cre^+^ mice) and Cre-negative littermate controls (SOCS3^fl/fl^ MCK-Cre^−^ mice). All experimental protocols were approved by the Animal Ethics Committee of The University of Melbourne and conducted in accordance with the Australian code of practice for the care and use of animals for scientific purposes as stipulated by the National Health and Medical Research Council (Australia).

To confirm SOCS3 gene deletion by genomic PCR, DNA was extracted from liver and skeletal muscle tissue from SOCS3^fl/fl^ MCK-Cre^+^ and SOCS3^fl/fl^ MCK-Cre^−^ mice using the GenElute genomic DNA miniprep (Sigma-Aldrich, St. Louis, MO, USA) as per manufacturer’s instructions. Genomic PCR was performed using the following forward and reverse primer sequences: SOCS3, 5′-ACGTCTGTGATGCTTTGCTG-3′ and 5′-TCTTGTGTCTCTCCCCATCC-3′, under the following conditions: step 1—94 °C 3 min, step 2—94 °C 30 s, step 3—55 °C 30 s, step 4—72 °C 60 s, cycle steps 2–4 36 times, step 5—72 °C 5 min.

To confirm SOCS3 deletion in muscle fibers, SOCS3^fl/fl^ MCK-Cre^+^ and SOCS3^fl/fl^ MCK-Cre^−^ mice received an intraperitoneal injection (i.p.) of saline or lipopolysaccharide (LPS 1 mg/kg; Sigma-Aldrich) and were killed 4 h post-injection. Gastrocnemius muscles were removed and snap frozen. Muscles were freeze-dried overnight and freed from visible adipose and connective tissue, and individual muscle fibers were dissected and placed into clean Eppendorf tubes. Total RNA was extracted using an RNeasy Fibrous Tissue Mini Kit (Qiagen, Venlo, Limburg, Netherlands) as per manufacturer’s instructions. The concentration and quality of RNA samples were determined using Nanodrop 2000 spectrophotometer (Thermo Scientific, Waltham, MA, USA). Real-time RT-PCR was performed as described previously [30] using the following forward and reverse primer sequences: SOCS3, 5′-GCTGGCCAAAGAAATAACCA-3′ and 5′- AGCTCACCAGCCTCATCTGT-3′. Relative gene expression was calculated using the expression 2^−ΔCT^, normalized to total complementary DNA (cDNA) content as determined using Qubit 2.0 Fluorometer (Life Technologies, Carlsbad, CA, USA), as described previously [12].

### Myotoxic injury

Twelve-week-old or 24-month-old male and female SOCS3 MKO (SOCS3^fl/fl^ MCK-Cre^+^) mice and their littermate controls (SOCS3^fl/fl^ MCK-Cre^−^ mice) were anesthetized with an intraperitoneal injection (i.p.) of 100 mg/kg ketamine (Ceva Animal Health Pty. Ltd., Glenorlie, NSW, Australia) and 10 mg/kg xylazine (ilium xylazil-20; Troy Laboratories, Smithfield, NSW, Australia). Once non-responsive to tactile stimuli, the right tibialis anterior (TA) muscle was surgically exposed and injected with 40 μl notexin (10 μg/ml saline; Latoxan, Valence, France) to induce muscle fiber degeneration. After the intramuscular injection, the skin incision was closed with Michel clips (Aesculap, Tuttlingen, Germany), and the mouse allowed to recover on a heating pad. In 12-week-old mice, the right TA muscle was excised and weighed at 1, 2, 3, 5, 7, or 14 days post-notexin injury. In 24-month-old mice, the right TA muscle was excised and weighed at 7 days post-notexin injury only. The right TA muscles were also excised from uninjured mice for use as controls. The right TA was cut transversely with one third immediately snap frozen in liquid nitrogen for RNA extraction and the remaining two thirds coated with optimal cutting temperature (OCT) compound (Tissue-Tek, Sakura Finetek, CA, USA) and frozen in thawing isopentane for histological analyses and western immunoblotting. All muscles were subsequently stored at −80 °C.

### Assessment of contractile properties of skeletal muscle and tissue collection

At 7 days post-notexin injury, both injured and uninjured mice were anesthetized with sodium pentobarbitone (Nembutal; 60 mg/kg; Sigma-Aldrich) via i.p. injection. The methods for assessment of the contractile properties of the mouse tibialis anterior muscle in situ have been described in detail previously [31]. At the conclusion of the contractile measurements in situ, the right and left TA muscles were carefully excised, blotted on filter paper, weighed on an analytical balance, and frozen in thawing isopentane for later histological examination.

### Skeletal muscle histology

Serial sections (5 μm) were cut transversely through the TA muscle using a refrigerated (−20 °C) cryostat (CTI Cryostat; IEC, Needham Heights, MA). Sections were stained (or reacted) with hematoxylin and eosin (H&E) to determine general muscle architecture, an anti-laminin antibody (#L9393; Sigma-Aldrich, St. Louis, MO, USA) for determination of mean myofiber CSA, or succinate dehydrogenase (SDH) to determine activity of oxidative enzymes [32]. Optical density (OD) of SDH was determined after 6 min of reactivity for all samples, and SDH-reacted sections were captured in full color using bright-field light microscopy and analyzed, as described previously [31]. For histological assessments and analysis of right TA muscle CSA in aged mice, comparisons were made to a small number of older uninjured control and SOCS3 MKO mice aged between 17 and 24 months of age (*n* = 5/genotype). Digital images of stained sections were obtained using an upright microscope with camera (Axio Imager day 1, Carl Zeiss, Wrek, Göttingen, Germany), controlled by AxioVision AC software (AxioVision AC Rel. 4.8, Carl Zeiss Imaging Solutions, Wrek, Göttingen, Germany). Images were quantified using AxioVision 4.8.2 software.

### Skeletal muscle immunostaining

Serial sections (5 μm) were cut transversely through the TA muscle using a refrigerated (−20 °C) cryostat (CTI Cryostat; IEC, Needham Heights, MA). Sections were fixed for 10 min in methanol at −20 °C, air-dried, and subsequently incubated with Alexa488- and Alexa647-conjugated antibodies raised against F4/80 and CD68 (Abcam., Cambridge, UK), respectively, for 1 h at room temperature in a humidified chamber. Slides were rinsed for 5 min in PBS containing 0.05 % Tween20 (PBStw) and 2 × 5 min in PBS and then incubated for 30 min with 4′,6-diamindino-2-phenylindole (DAPI, 5 μg/mL PBS) to visualize nuclei. After washing with PBStw and PBS, the sections were embedded in Mowiol® and covered with a coverslip. Digital images of stained sections were obtained using an upright microscope with camera (Axio Imager D1, Carl Zeiss, Wrek Göttingen, Germany), controlled by AxioVision AC software (AxioVision AC Rel. 4.8.2, Carl Zeiss Imaging Solutions, Wrek, Wrek Göttingen, Germany) as described previously.

### RNA extraction and qPCR

Total RNA was extracted from each portion of right TA muscle (*n* = 6/genotype/time-point) using an RNeasy Fibrous Tissue Mini Kit (Qiagen, Venlo, Limburg, Netherlands) as per manufacturer’s instructions. The concentration and quality of RNA samples was determined using Nanodrop 2000 spectrophotometer (Thermo Scientific, Waltham, MA, USA). Real-time RT-PCR was performed as described previously [30] using the following forward and reverse primer sequences: MCK, 5′-CACCATGCCGTTCGGCAACA-3′ and 5′-GGTTGTCCACCCCAGTCT-3′; SOCS3, 5′-GCTGGCCAAAGAAATAACCA-3′ and 5′-AGCTCACCAGCCTCATCTGT-3′; Pax7, 5′-GGAAAACCAGTGTGCCATCT-3′ and 5′-CCTTGTCTTTGGCACCATTT-3′; MyoD, 5′-AGTGAATGAGGCCTTCGAGA-3′ and 5′-GCATCTGAGTCGCCACTGTA-3′; Myogenin, 5′-CACTCCCTTACGTCCATCGT-3′ and 5′-CAGGACAGCCCCACTTAAAA-3′; IL-6, 5′-CCGGAGAGGAGACTTCACAG-3′ and 5′-TCCACGATTTCCCAGAGAAC-3′; TNF-α, 5′-GGCCTTCCTACCTTCAGACC-3′ and 5′-AGCAAAAGAGGAGGCAACAA-3′; IFN-γ, 5′-ACTGGCAAAAGGATGGTGAC-3′ and 5′-TGAGCTCATTGAATGCTTGG-3′; F4/80, 5′-CATCAGCCATGTGGGTACAG-3′ and 5′-CATCACTGCCTCCACTAGCA-3′; CD68, 5′-TCCAAGCCCAAATTCAAATC-3′ and 5′-ATTGTATTCCACCGCCATGT-3′. Gene expression was quantified using a cycle threshold (C_T_) method. As myotoxic injury with notexin causes complete degradation of skeletal muscle fibers, normalizing mRNA to a housekeeping gene is difficult, and so relative gene expression was calculated using the expression 2^−ΔCT^, normalized to total cDNA content as determined using Qubit 2.0 Fluorimeter (Life Technologies, Carlsbad, CA, USA), as described previously [12].

### Antibodies

The following primary antibodies were used throughout the experiments in 5 % BSA/TBS/0.1 % Tween-20: rabbit-anti-phosphorylated STAT1 (Y701) (Cell Signaling Technology, Danvers, MA, USA; 1:1000), mouse-anti-STAT1 (BD Biosciences, San Jose, CA, USA; 1:1000), rabbit-anti-phosphorylated STAT3 (Y705) (Cell Signaling Technology, 1:1000), rabbit-anti-STAT3 (Cell Signaling Technology, 1:1000), and rabbit-anti-SOCS3 (Immunobiological Laboratories Co. Ltd., Gunma, Japan; 1:200). Horseradish peroxidase (HRP)-conjugated donkey-anti-rabbit immunoglobulin (GE healthcare life sciences; Marlborough, MA, USA) secondary antibody was used at 1:5000 in 5 % BSA/TBS/0.1 % Tween-20. HRP-conjugated sheep-anti-mouse immunoglobulin (GE Healthcare Life Sciences) secondary antibody was used at 1:5000 in 5 % BSA/TBS/0.1 % Tween-20.

### Western immunoblotting

For protein analysis from C2C12 cells, media was aspirated at the conclusion of treatment and attached cells were lysed directly in the plate on ice in cold RIPA buffer (50 mM Tris–HCl, 150 mM NaCl, 1 mM EDTA, 1 % Triton X-100, 1 % Na-Deoxycholate, 0.1 % SDS, protease inhibitor cocktail) for 10 min. Lysates were collected into Eppendorf tubes and diluted in 4× Lammeli sample buffer (0.25 M Tris-HCl, pH6.8, 6 % SDS, 40 % glycerol, 0.04 % bromophenol blue, 16 % DTT), run on 4–15 % Criterion TGX Stain-Free gels (Bio-Rad Laboratories, Gladesville, NSW, Australia) at a constant voltage of 100 V and transferred to PVDF membranes by wet transfer.

For protein analysis from muscle tissues, the remaining OCT embedded right TA muscle was excised from the OCT media with a razor blade following sectioning. Excised muscles were homogenized in metal bead-containing matrix tubes in cold RIPA buffer (50 mM Tris-HCl, 150 mM NaCl, 1 mM EDTA, 1 % Triton X-100, 1 % Na-Deoxycholate, 0.1 % SDS, protease inhibitor cocktail) using a Precellys24 automated homogenizer. Homogenized muscle samples were diluted in 3× SDS loading buffer (125 mM Tris-HCl, 4 M urea, 10 % glycerol, 4 % SDS, 10 % β-ME, and 0.001 % bromophenol blue) according to muscle wet concentration and run on 4–15 % Criterion TGX Stain-Free gels (Bio-Rad Laboratories) at a constant voltage of 200 V and transferred to PVDF membranes using the Trans-Blot Turbo system (Bio-Rad Laboratories).

Membranes were blocked in 5 % BSA in TBST and incubated at 4 °C overnight in primary antibody solutions. Horseradish peroxidase (HRP)-conjugated secondary antibodies were applied for 1 h at RT. Membranes were visualized with ECL (SuperSignal West Femto Chemiluminescent Substrate, Thermo Scientific Pierce, IL, USA) and imaged using ChemiDoc MP Imaging System. A stain-free image of each gel was acquired prior to transfer. ECL and stain-free images were quantified using Image Lab 4.1 software (Bio-Rad Laboratories). The intensity of each protein of interest was normalized to the total protein content of the sample as determined from the stain-free image as described previously [33].

### Statistical analyses

For in vitro studies, data were analyzed between groups using a one-way analysis of variance (ANOVA) with a Fisher’s LSD post hoc multiple comparisons test for comparison to untreated cells. For all in vivo work, data were analyzed between groups for the effect of genotype and time using a two-way ANOVA with Fisher’s LSD post hoc multiple comparisons test used to detect significant differences between means where appropriate. A *P* value less than 0.05 was considered statistically significant. All statistical analyses were carried out using Prism Graphpad 6 software (GraphPad Software Inc., La Jolla, CA, USA). All values are presented as mean ± standard error of mean (SEM).

## Results

### Inflammatory cytokines induce JAK/STAT signaling and SOCS3 protein in myoblasts and myotubes in vitro

**Fig. 1:**
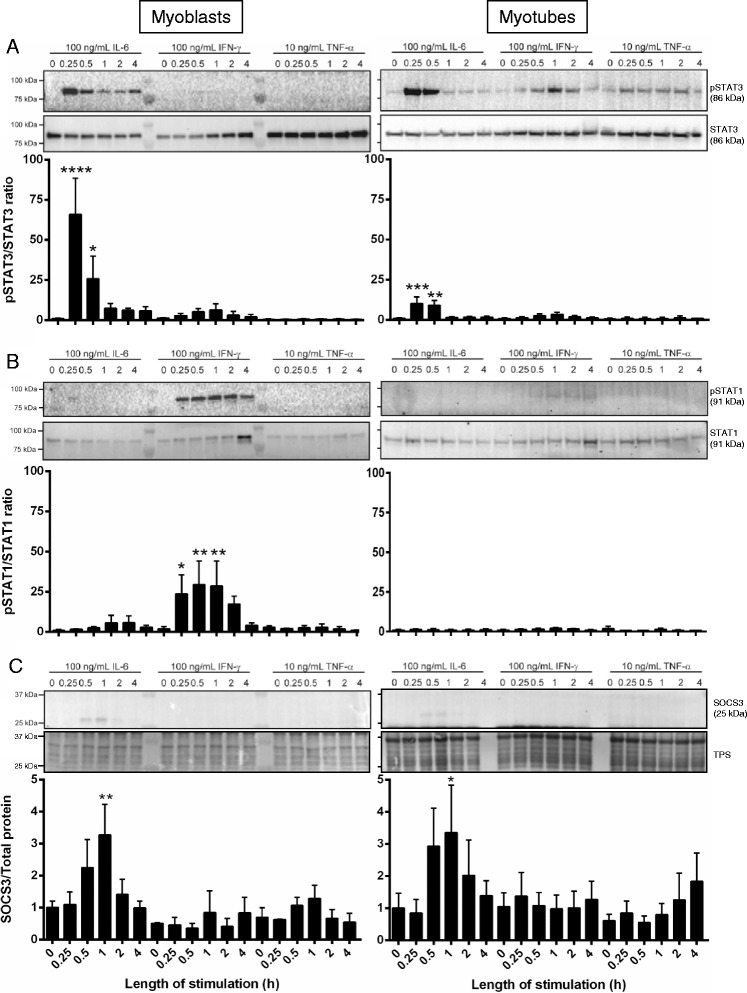
IL-6 and IFN-γ stimulation initiate JAK/STAT signaling in C2C12 myoblasts and myotubes in vitro. C2C12 proliferating myoblasts (*left*) or differentiated day 5 myotubes (*right*) either remained unstimulated (0) or were incubated with 100 ng/mL rmIL-6, 100 ng/mL rmIFN-γ, or 10 ng/mL rmTNF-α for 0.25, 0.5, 1, 2, or 4 h at 37 °C + 5 % CO_2_. At the conclusion of stimulation, cells were lyzed and subject to SDS-PAGE and western immunoblotting for either pSTAT3/STAT3 (**a**), pSTAT1/STAT1 (**b**), or SOCS3 and total protein (TPS; **c**). Representative immunoblots are shown for each probe with *n* = 1 per time-point. Three individual experiments were performed, and bands were quantified to produce the graphs shown. Data are expressed as mean ± SEM. Statistical analysis was performed using a one-way ANOVA with a Fisher’s LSD post hoc multiple comparisons test to determine the effect of treatment. *P < 0.05, **P < 0.01, ***P < 0.001, ****P < 0.0001 compared to unstimulated cells

The pro-inflammatory cytokines IL-6, IFN-γ, and TNF-α induce JAK/STAT signaling and are expressed at high levels after myotoxic injury [34–36]. The signaling pathways induced by these cytokines specifically within muscle cells were examined in C2C12 myoblasts and myotubes in vitro. While no STAT3 phosphorylation was observed in unstimulated C2C12 myoblasts (0 h), a low basal level of phosphorylated STAT3 was present in unstimulated C2C12 myotubes (0 h; Fig. 1a). Addition of rm IL-6 (100 ng/mL) significantly increased STAT3 phosphorylation after 15 min stimulation in both myoblasts and myotubes which decreased at 1 h stimulation (Fig. 1a). In comparison, stimulation with rm IFN-γ (100 ng/mL) induced very low levels of STAT3 phosphorylation in both myoblasts and myotubes, and rm TNF-α stimulation (10 ng/mL) did not induce phosphorylation of STAT3 in either cell population (Fig. 1a). Phosphorylation of STAT1 occurred within 15 min and up to 4 h of stimulation with rm IFN-γ in C2C12 myoblasts and to a lesser extent in C2C12 myotubes (Fig. 1b). No significant STAT1 phosphorylation was observed following rm IL-6 or rm TNF-α stimulation in either cell type (Fig. 1b).

STAT3 dimerizes after phosphorylation and translocates to the nucleus where it regulates the transcription of many genes, including SOCS3, which in turn negatively regulates the activation of STAT3 [37]. SOCS3 protein was increased after 30 min and 1 h of rm IL-6 stimulation, but not rm IFN-γ or rm TNF-α stimulation, in myoblasts or myotubes (Fig. 1c). Together, these data demonstrate that inflammatory cytokines, particularly IL-6, activate signaling pathways directly within skeletal muscle cells, resulting in production of SOCS3 protein.

### SOCS3 deletion specifically within mature muscle fibers does not alter the regenerative response after myotoxic injury

As our in vitro data demonstrated that JAK/STAT signaling induced SOCS3 protein expression in both skeletal muscle myoblasts and mature myotubes, we utilized the SOCS3^fl/fl^ MCK-Cre mouse, in which the *Socs3* gene is deleted only in cells expressing muscle creatine kinase (MCK), to determine whether absence of SOCS3 in mature skeletal muscle fibers impairs muscle fiber regeneration. The specificity of the MCK-Cre mediated SOCS3 deletion was confirmed by genomic PCR which showed the presence of the 288 bp deleted SOCS3 fragment in skeletal muscles but not livers of SOCS3 MKO mice (Additional file 1: Figure S1A, B) and by qPCR which demonstrated a lipopolysaccharide (LPS)-induced increase in *Socs3* gene expression in isolated fibers from freeze-dried gastrocnemius muscles from control but not SOCS3 MKO mice (Additional file 1: Figure S1C).

**Fig. 2:**
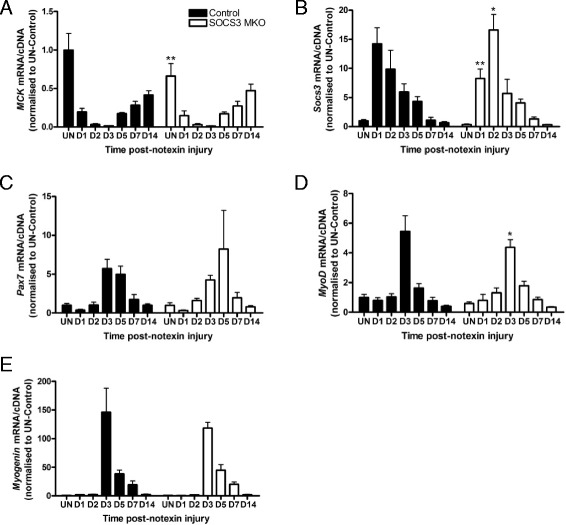
SOCS3 deletion in mature myofibers in vivo does not alter regeneration after myotoxic injury. Control (SOCS3^fl/fl^ MCK-Cre^−^) and SOCS3 MKO (SOCS3^fl/fl^ MCK-Cre^+^) mice were either left uninjured (UN) or received a single 40 μL injection of notexin (10 μg/ml) into the right TA muscle and were killed for analysis at 1 day (D1), 2 days (D2), 3 days (D3), 5 days (D5), 7 days (D7), or 14 days (D14) post-notexin injury. qRT-PCR using primers to detect *MCK* (**a**), *Socs3* (**b**), *Pax7* (**c**), *MyoD* (**d**), and *Myogenin* (**e**) was performed on RNA extracted from snap frozen muscles following dissection. Data are expressed as mean ± SEM. Statistical analysis was performed using a two-way ANOVA with a Fisher’s LSD post hoc multiple comparisons test to determine the effects of genotype and time. *n* = 8 mice/time-point/genotype. *P < 0.05, **P < 0.01 compared to control

To determine the time-points after myotoxic injury in which SOCS3 deletion would be expected, we examined the gene expression of *MCK*. In both control and SOCS3 MKO mice, *MCK* gene expression was detected in uninjured muscle (UN), decreased at day 1, day 2, and day 3 and subsequently increased progressively from day 5 to day 14 post-notexin injury (Fig. 2a; *P* < 0.0001 injury main effect). However, *MCK* expression was lower in uninjured muscles from SOCS3 MKO mice relative to control (Fig. 2a; **P < 0.01). *Socs3* gene expression was detected at low levels in uninjured muscles and was not different between muscles from control and SOCS3 MKO mice (Fig. 2b). In control mice, *Socs3* gene expression increased at day 1 post-notexin injury and decreased progressively to basal levels by day 14 (Fig. 2b; *P* < 0.0001 injury main effect). The increase in *Socs3* gene expression after notexin injury was delayed in muscles from SOCS3 MKO mice, being lower at day 1 (**P < 0.01) and higher at day 2 (*P < 0.05) compared to control but decreased to basal levels by day 14 (Fig. 2b; *P* < 0.0001 injury main effect). Therefore, deletion of *Socs3* in MCK-expressing mature muscle fibers alters the expression profile of the *Socs3* gene after myotoxic injury.

To examine the progression of muscle regeneration post-injury, we analyzed the gene expression of the muscle stem cell marker, *Pax7* (Fig. 2c), the master myogenic regulator, *MyoD* (Fig. 2d), and the marker of early muscle cell differentiation, *Myogenin* (Fig. 2e). In muscles from both control and SOCS3 MKO mice, the expression of all three markers was low in uninjured muscles and at day 1 and day 2 post-notexin injury, peaked at day 3, and subsequently decreased to basal levels by day 14 (Fig. 2c–e; *P* < 0.001 injury main effect). While *MyoD* expression was decreased slightly at day 3 post-notexin injury in muscles from SOCS3 MKO mice relative to control (Fig. 2d; *P < 0.05), the time-course of *Pax7*, *MyoD*, and *Myogenin* gene expression did not vary compared with control indicating that regeneration was not altered by muscle fiber-specific SOCS3 deletion.

### Loss of SOCS3 in skeletal muscle fibers increases the early inflammatory response after myotoxic injury

**Fig. 3:**
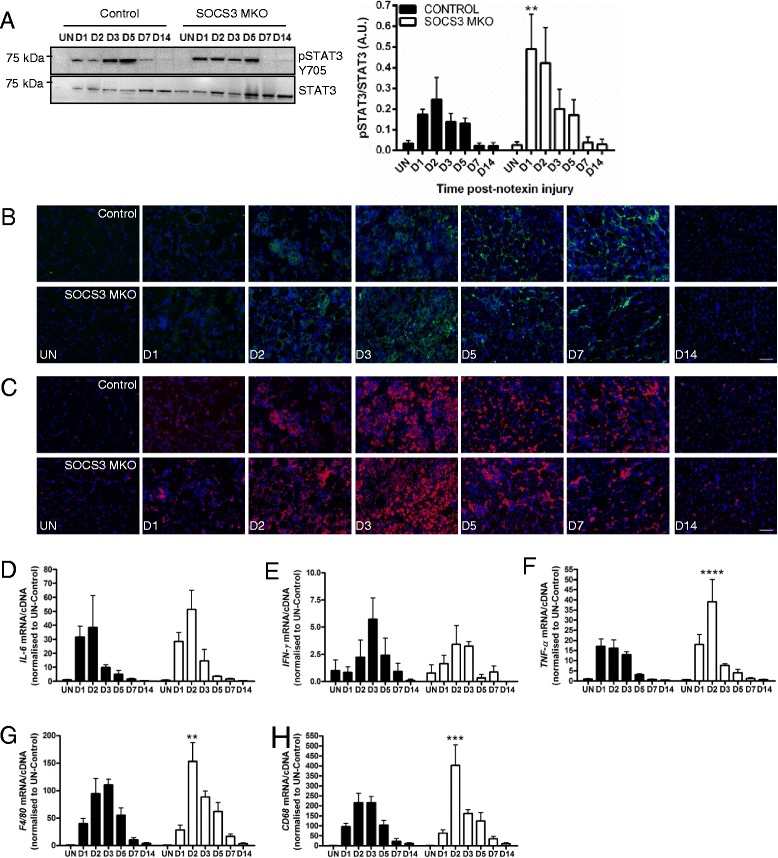
Loss of SOCS3 in mature myofibers in vivo enhances the inflammatory response after myotoxic injury. Control (SOCS3^fl/fl^ MCK-Cre^−^) and SOCS3 MKO (SOCS3^fl/fl^ MCK-Cre^+^) mice were either left uninjured (UN) or received a single 40 μL injection of notexin (10 μg/ml) into the right TA muscle and then killed for analysis at 1 day (D1), 2 days (D2), 3 days (D3), 5 days (D5), 7 days (D7), or 14 days (D14) post-notexin injury. **a** Western immunoblotting for phosphorylated and total STAT3 protein levels was performed on protein extracted from remaining OCT embedded muscles following tissue sectioning. Representative immunoblots for phosphorylated (*top*) and total (*bottom*) STAT3 protein levels are shown. Band intensity was quantified using ImageQuant software (Bio-Rad Laboratories) and the ratio of phosphorylated/total STAT3 protein levels was determined. **b** Representative sections immunostained with F4/80 (*green*) and DAPI (*blue*) of the TA muscle from uninjured or day 1, 2, 3, 5, 7, or 14 injured control and SOCS3 MKO mice. **c** Representative sections immunostained with CD68 (*red*) and DAPI (*blue*) of the TA muscle from uninjured or day 1, 2, 3, 5, 7, or 14 injured control and SOCS3 MKO mice. qRT-PCR using primers to detect *IL-6* (**d**), *IFN-γ* (**e**), *TNF-α* (**f**), *F4/80* (**g**), and *CD68* (**h**) was performed on RNA extracted from snap frozen muscles following dissection. Data are expressed as mean ± SEM. Statistical analysis was performed using a two-way ANOVA with a Fisher’s LSD post hoc multiple comparisons test to determine the effects of genotype and time. *n* = 8 mice/time-point/genotype. **P < 0.01, ***P < 0.001, ****P < 0.0001 compared to control. *Scale bar* = 50 μm

We examined the effect of SOCS3 deletion on the activation of JAK/STAT signaling during regeneration after myotoxic injury. No phosphorylation of JAK1, JAK2, STAT1, or STAT5 was detected in muscle lysates from uninjured control or SOCS3 MKO mice (data not shown). In control and SOCS3 MKO mice, STAT3 phosphorylation was not detected in uninjured muscles but was increased at day 1 post-notexin injury and remained constant at days 2, 3, and 5 then decreased to basal levels by day 7 (Fig. 3a; *P* < 0.0001 injury main effect). In muscles from SOCS3 MKO mice, STAT3 phosphorylation was significantly increased compared to muscles from control mice at day 1 post-notexin injury (Fig. 3a; **P < 0.01 compared to control).

As increased STAT3 phosphorylation is an indicator of an increased inflammatory response, we next examined the presence of inflammatory cells and the gene expression of inflammatory markers. Immunostaining for the inflammatory cell markers F4/80 (Fig. 3b) and CD68 (Fig. 3c) showed increased presence of these cells at D2 and D3 following injury in muscles from control and SOCS3 MKO mice and indicated an increased infiltration of inflammatory cells at D2 following injury in muscles from SOCS3 MKO mice compared to control (Fig. 3b, c). The inflammatory cytokine *IL-6* gene was expressed at low levels in uninjured muscles, highly expressed at day 1 and day 2 post-injury and decreased to basal levels by day 14 (Fig. 3d; *P* < 0.0001 injury main effect). Gene expression of *IFN-γ* increased around day 3 post-notexin injury and was barely detected by day 14 (Fig. 3e; *P* < 0.01 injury main effect). SOCS3 deletion had no effect on *IL-6* (Fig. 3d) or *IFN-γ* (Fig. 3e) expression after myotoxic injury.

The gene expression of the pro-inflammatory cytokine *TNF-α*, as well as the inflammatory cell markers *F4/80* and *CD68*, was low in uninjured muscles from control and SOCS3 MKO mice, increased progressively to day 3, then reduced to basal levels by day 14 post-notexin injury (Fig. 3f–h; *P* < 0.0001 time main effect). In muscles from SOCS3 MKO mice, *TNF-α*, *F4/80* and *CD68* gene expression was higher compared to muscles from control mice at day 2 post-notexin injury (Fig. 3f–h; ****P < 0.0001, **P < 0.01, ***P < 0.001, respectively). Together, these results suggest that muscle fiber-specific SOCS3 deletion increases the inflammatory signal at day 1, resulting in an enhanced inflammatory response at day 2, which appears resolved by day 3 post-notexin injury.

### Skeletal muscle fiber-specific deletion of SOCS3 does not alter force production before or after myotoxic injury

**Fig. 4:**
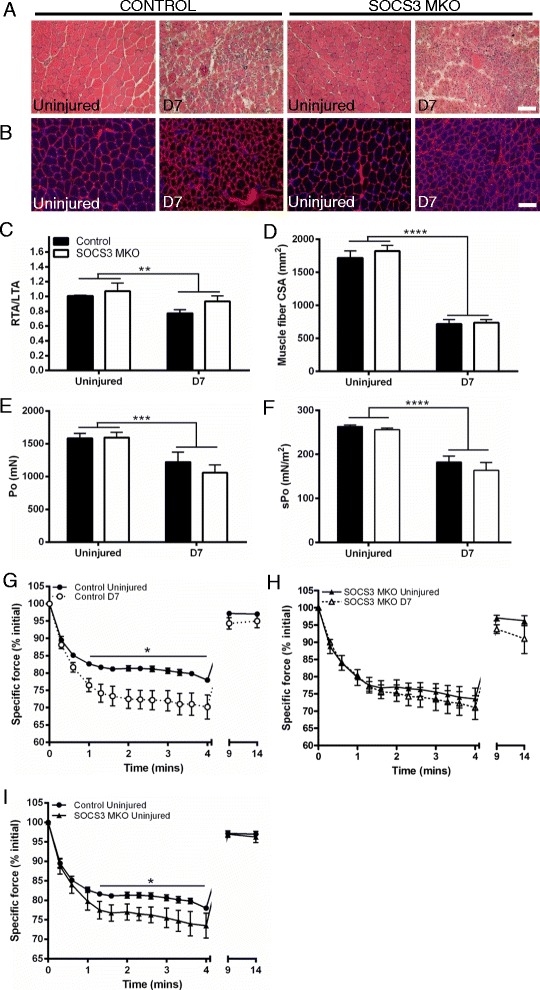
Skeletal muscle fiber-specific deletion of SOCS3 does not alter force production of the TA muscles. Control (SOCS3^fl/fl^ MCK-Cre^−^) and SOCS3 MKO (SOCS3^fl/fl^ MCK-Cre^+^) mice were either left uninjured (UN) or received a single 40 μL injection of notexin (10 μg/ml) into the right TA muscle. Representative hematoxylin and eosin (**a**) and laminin and DAPI (**b**) stained sections of TA muscle from uninjured or day 7 injured control and SOCS3 MKO mice. Muscle mass relative to the uninjured left TA muscle (**c**), muscle fiber size (**d**), maximum isometric force (**e**), and specific (normalized) force (**f**) were determined at day 7 post-notexin injury. Data are expressed as mean ± SEM. Statistical analysis was performed using a two-way ANOVA with a Fisher’s LSD post hoc multiple comparisons test to determine effects of genotype and time. *n* = 8 mice/time-point/genotype. **﻿*P* < 0.01, ***P < 0.001, ****P < 0.0001 compared to uninjured muscle. Specific force production during a 4-min fatiguing protocol comparing uninjured and day 7 injured right TA muscles from control mice (**g**), SOCS3 MKO mice (**h**), and comparing uninjured control and SOCS3 MKO mice separately (**i**). Data are expressed as mean ± SEM. Statistical analysis was performed using a repeated measures two-way ANOVA with a Fisher’s LSD post hoc multiple comparisons test to determine effects of genotype and time. *n* = 8 mice/time-point/genotype. *P < 0.05 compared to uninjured muscle from control mice. *Scale bar* = 50 μm

To determine whether an enhanced early inflammatory response after myotoxic injury in muscles lacking SOCS3 had any effect on muscle function, we next examined mass and force production of uninjured and notexin-injured TA muscles from control and SOCS3 MKO mice. Histology of uninjured muscles from aged control and SOCS3 MKO mice appeared normal and ongoing repair was observed in injured muscles as indicated by the continued presence of centrally-nucleated fibers, which looked similar in SOCS3 MKO muscles (Fig. 4a, b). Skeletal muscle-specific deletion of SOCS3 did not affect the mass of the uninjured right TA muscle or at 7 days post-notexin injury, but control and SOCS3 MKO muscles were smaller after notexin injury (Fig. 4c; *P* < 0.01 injury main effect). Similarly, average muscle fiber cross-sectional area (CSA) was reduced at 7 days post-injury compared with uninjured muscles and was not changed with SOCS3 deletion (Fig. 4d; *P* < 0.0001 injury main effect). Both maximum isometric force and specific force were reduced 7 days after notexin injury compared with uninjured muscles and were similarly not affected by SOCS3 deletion (Fig. 4e, f; *P* < 0.0001 injury main effect). SOCS3 deletion did not affect force production at any stimulation frequency in either the uninjured muscles (Additional file 2: Figure S2A) or in muscles at 7 days post-injury (Additional file 2: Figure S2B). Notexin injury reduced force production during a 4-min fatiguing protocol in control mice (Fig. 4g; *P* < 0.05 compared with uninjured muscles), but there was no difference in the fatigue response between uninjured and injured muscles of SOCS3 MKO mice (Fig. 4h). Analysis of force production over the 4-min fatiguing protocol between uninjured muscles from control and SOCS3 MKO mice showed this difference to be due to an increased fatigue response in the uninjured muscles of SOCS3 MKO mice (Fig. 4i; *P < 0.05 compared to control). Gene expression analysis showed no significant change in expression of myosin heavy chain isoforms *MyHCIIb* (Additional file 3: Figure S3A), *MyHCIIx* (Additional file 3: Figure S3B), *MyHCI* (Additional file 3: Figure S3C), or *MyHCIIa* in muscles from SOCS3 MKO mice (Additional file 3: Figure S3D), and oxidative capacity of muscles from SOCS3 MKO was unchanged compared to control (Additional file 3: Figure S3E).

### Loss of SOCS3 does not alter regeneration or inflammation after myotoxic injury in aged mice

**Fig. 5:**
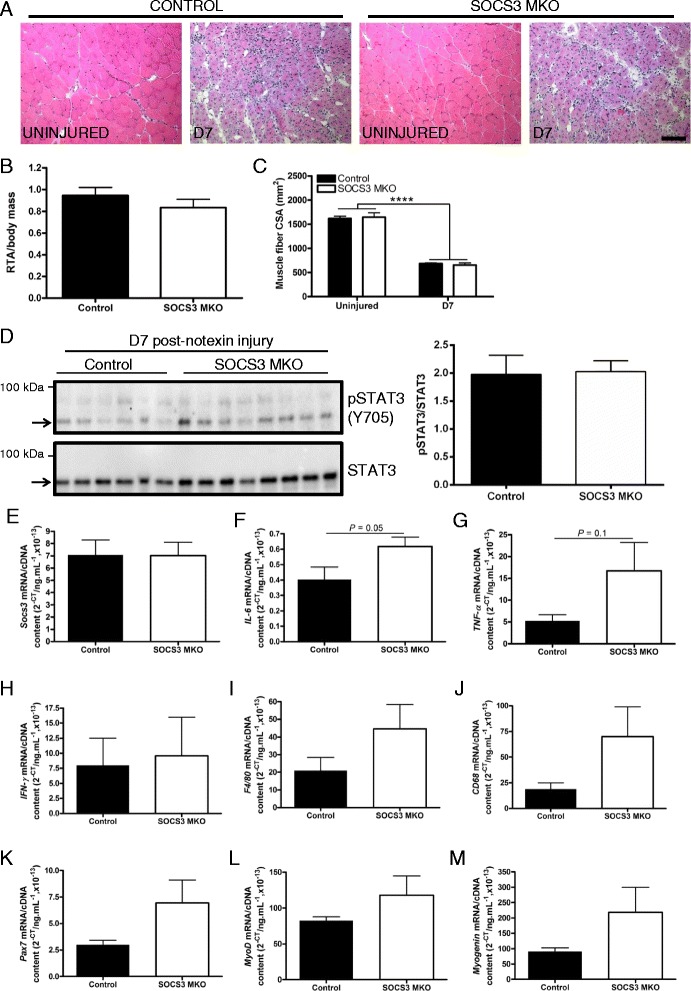
SOCS3 deletion does not alter regeneration or inflammation after myotoxic injury in aged mice. Twenty-four-month-old control (SOCS3^fl/fl^ MCK-Cre^−^) and SOCS3 MKO (SOCS3^fl/fl^ MCK-Cre^+^) mice were either left uninjured (UN) or received a single 40 μL injection of notexin (10 μg/ml) into the right TA muscle. **a** Representative hematoxylin and eosin-stained sections of the TA muscle from uninjured or day 7 injured control and SOCS3 MKO mice. Muscle mass relative to body mass (**b**) and muscle fiber size (**c**) was determined at day 7 post-notexin injury. **d** Western immunoblotting for phosphorylated and total STAT3 protein levels was performed on protein extracted from the remaining OCT embedded muscles following tissue sectioning. Representative immunoblots for phosphorylated (*top*) and total (*bottom*) STAT3 protein levels are shown. Band intensity was quantified using ImageQuant software (Bio-Rad Laboratories), and the ratio of phosphorylated/total STAT3 protein levels was determined. Bands used for quantification are indicated by *arrows*. qRT-PCR using primers to detect *Socs3* (**e**), *IL-6* (**f**), *TNF-α* (**g**), *IFN-γ* (**h**), *F4/80* (**i**), *CD68* (**j**), *Pax7* (**k**), *MyoD* (**l**), or *Myogenin* (**m**) was performed on RNA extracted from snap frozen muscles following dissection. Data are expressed as mean ± SEM. Statistical analysis was performed using an unpaired two-tailed Student’s *t* test (*n* = 7–8 mice/time-point/genotype) except for analysis of muscle fiber size (**c**) which was analyzed with a two-way ANOVA and Fisher’s LSD post hoc multiple comparisons test to determine the effect of genotype and injury (*n* = 4–8 mice/time-point/genotype). *Scale bar* = 100 μm

As skeletal muscle-specific SOCS3 deletion models reduced SOCS3 levels and lower SOCS3 levels have been reported in muscles from aged mice [29], we next examined whether loss of SOCS3 in skeletal muscles impacted on muscle regeneration in aged (24 month old) mice. Notexin injuries were performed on 24-month-old control and SOCS3 MKO mice (*n* = 7–8/genotype) and markers of regeneration and inflammation were assessed in the injured muscles at 7 days post-injury. Histology of uninjured muscles from aged control and SOCS3 MKO mice appeared normal, but consistent with previous reports, ongoing inflammation and repair was observed in injured 24-month-old control muscles, which looked similar in SOCS3 MKO muscles (Fig. 5a). At 7 days post-notexin injury, SOCS3 deletion did not alter TA mass relative to body mass (Fig. 5b), and while muscle fiber CSA was significantly smaller in injured muscles, SOCS3 deletion had no further effect (Fig. 5c; ****P < 0.0001 injury main effect).

Western immunoblotting revealed no difference in STAT3 phosphorylation in SOCS3 MKO muscles compared to control (Fig. 5d), and no change was observed in *Socs3* RNA levels between control and SOCS3 MKO mice at 7 days post-notexin injury, likely due to the continued expression of *Socs3* by muscle resident cells other than the myofibers (Fig. 5e). While no significant changes were observed, gene expression levels of the inflammatory cytokines *IL-6* (Fig. 5f; *P* = 0.05) and *TNF-α* (Fig. 5g; *P* = 0.1) but not *IFN-γ* (Fig. 5h; *P* = 0.84) or the inflammatory cell markers *F4/80* (Fig. 5i; *P* = 0.19) or *CD68* (Fig. 5j; *P* = 0.16) tended to be increased in muscles from SOCS3 MKO mice compared to control at 7 days post-notexin injury. Similarly, gene expression levels of the myogenic markers *Pax7* (Fig. 5k; *P* = 0.15), *MyoD* (Fig. 5l; *P* = 0.28), and *Myogenin* (Fig. 5m; *P* = 0.21) were not changed in muscles of SOCS3 MKO mice compared with control at 7 days post-injury.

## Discussion

SOCS3 RNA and/or protein levels are altered in muscles with aging [27–29] and may be implicated in impaired muscle regeneration with aging; but whether they increase or decrease remains to be determined. SOCS3 is expressed by multiple cell types in regenerating skeletal muscles, but the relative contribution of SOCS3 within each cell type to altered muscle inflammation and regeneration is unknown. Using mice specifically lacking SOCS3 in MCK-expressing mature muscle fibers, we have shown that although reduced SOCS3 expression increases the early inflammatory response in skeletal muscle after myotoxic injury, this does not impact regeneration.

SOCS3 MKO mice undergo normal muscle development and exhibit improved insulin sensitivity when fed a high fat diet compared to control mice [26]. Consistent with these findings, we observed no significant changes in muscle structure and function between injured and uninjured muscles of control and SOCS3 MKO mice. However, uninjured TA muscles from SOCS3 MKO mice were more susceptible to fatigue than muscles from control mice, in the absence of any changes in oxidative capacity or MyHC isoform gene expression. Treadmill running performance was unchanged between control and SOCS3 MKO mice [26], indicating that while muscle fiber-specific SOCS3 deletion increased fatigue in contracting TA muscles, overall exercise capacity was unaffected.

JAK/STAT signaling is intact in C2C12 cells in vitro, with addition of leukemia inhibitory factor (LIF), basic fibroblast growth factor (bFGF), growth hormone (GH), and leptin or insulin-like growth factor (IGF) all activating STAT3 [38–41]. The time-course of activation of STAT3 and induction of SOCS3 following addition of IL-6 to C2C12 cells was consistent with previous observations of the IL-6 family cytokine, LIF, in mouse embryonic stem (ES) cells, and the related cytokine granulocyte colony stimulating factor (G-CSF) in isolated mouse bone marrow cells [14, 42], indicating that muscle cells employ similar JAK/STAT signaling mechanisms to these other cell types. TNF-α and IFN-γ directly upregulate SOCS3 expression in hematopoietic cells and liver [37, 43–45], but we were unable to detect SOCS3 expression in C2C12 cells following stimulation with either IFN-γ or TNF-α. Endogenous SOCS3 protein expression is generally quite low and difficult to detect [46]. Therefore, if addition of TNF-α or IFN-γ did increase SOCS3 protein expression in C2C12 cells, this was below the level of detection and not to the level induced by IL-6 stimulation.

SOCS3 is a critical regulator of inflammatory signaling in many cell types. Conditional ablation of SOCS3 in various tissues, including hematopoietic cells and liver causes enhanced inflammation and emergency granulopoiesis [16, 17, 47–49]. Therefore, our observation of an increased inflammatory response after myotoxic damage in SOCS3 MKO mice was not unexpected. Increased inflammation could impact regeneration, but we observed no change in the regenerative capacity of muscles from SOCS3 MKO mice compared with control, likely because of the rapid resolution of the increased inflammatory cell infiltrate by D3 after injury.

SOCS3 is present in mature muscle fibers but also expressed in both muscle stem cells and inflammatory cells which are critical for successful muscle regeneration. We observed *Socs3* gene expression at varying levels after myotoxic injury in SOCS3 MKO mice indicating that SOCS3-expressing cells (most likely a combination of these two cell types) were present in the muscle at these times. SOCS3 expression is critical for the proper regulation of inflammatory cell function in multiple states of inflammation [14–17, 47, 48], and regulation of JAK/STAT signaling is critical to skeletal muscle stem cell function. Microarray studies have shown reduced *Socs3* gene expression upon muscle stem cell activation [19]. Furthermore, increased STAT3 activation has been reported in muscle stem cells isolated from muscles of old mice compared with those from younger mice [50] and reduction of STAT3 activation by either conditional ablation or transient pharmacologic inhibition modulates the proliferative and differentiation potential of the muscle stem cell pool [50, 51]. Therefore, SOCS3 expression and function is likely critical in the regulation of both the inflammatory response and muscle stem cell function in the context of muscle injury. We had hypothesized that reduced SOCS3 expression or function contributed to age-related impairments in muscle regeneration, but since regeneration was not impaired in SOCS3 MKO mice, reduced SOCS3 expression or function within the mature muscle fiber is unlikely to contribute directly. It remains possible that reduced SOCS3 protein expression within the muscle stem cell pool and/or inflammatory cells might impair the injury-repair process.

## Conclusions

Specific deletion of SOCS3 within MCK-expressing muscle fibers, which models the reduced SOCS3 expression thought to occur in muscles with aging, enhanced the early inflammatory response but ultimately did not affect muscle regeneration after myotoxic injury. Furthermore, inflammation and muscle regeneration were not altered after myotoxic injury in the muscles of 24-month-old SOCS3 MKO mice compared with control. Therefore, reduced SOCS3 expression/function within the muscle fiber is likely not a contributing factor to age-related impairments in muscle regeneration. However, since SOCS3 is expressed in other muscle resident cells, including hematopoietic cells and muscle stem cells, it remains possible that the expression level and/or function of SOCS3 may be altered in these populations with age. Further studies examining the effect of SOCS3 deletion in these populations on muscle regeneration are warranted.

## Additional files

Additional file 1: Figure S1. Genomic PCR to confirm correct *Socs3* deletion. (A) Schematic of *Socs3* gene demonstrating floxed regions (LoxP) flanking exon 2 and location of the sequencing primers both prior to and post-Cre excision. (B) Genomic PCR using the primers shown in (A) confirmed the presence of the 288 bp deleted DNA band in muscle but not liver of SOCS3^fl/fl^ MCK-Cre positive and not SOCS3^fl/fl^ MCK-Cre-negative mice. (C) qRT-PCR using primers to detect *Socs3* gene expression in RNA extracted from muscle fibers isolated from freeze-dried gastrocnemius muscles of saline or LPS-injected control and SOCS3 MKO mice. Data are expressed as mean ± SEM and compared with a two-way ANOVA and Fisher’s LSD post hoc multiple comparisons test to determine the effect of genotype and LPS injection (*n* = 2 mice/genotype). ****P* < 0.001 compared to uninjured muscle from control mice. (PDF 115 kb)
Additional file 2: Figure S2. Frequency-force relationships for uninjured and day 7 (D7) notexin-injured muscles from control and SOCS3 MKO mice. Frequency-force relations for specific (normalized) force at different stimulation frequencies (10–200 Hz) in muscles of either uninjured (A) or day 7 post-notexin-injured (B) control or SOCS3 MKO mice. Data are expressed as mean ± SEM. Comparisons were made using a two-way repeated measures ANOVA with Fisher’s LSD multiple comparisons test post hoc to determine the effect of genotype and stimulation frequency. *n* = 8 mice/genotype. (PDF 99 kb)
Additional file 3: Figure S3. Myosin heavy chain gene expression and muscle fiber oxidative capacity in muscles from uninjured control and SOCS3 MKO mice. qRT-PCR using primers to detect *MyHCIIb* (A), *MyHCIIx* (B), *MyHCI* (C), and *MyHCIIa* (D) was performed on RNA extracted from snap frozen muscles following dissection. Data are expressed as mean ± SEM and compared with an unpaired two-tailed Student’s *t* test. *n* = 8 mice/genotype. (E) Representative succinate dehydrogenase (SDH)-reacted TA muscle sections from uninjured muscles of 12-week-old control and SOCS3 MKO mice. Quantification of SDH intensity was determined by analysis of SDH reacted TA muscle sections. Data are expressed as mean ± SEM and compared with an unpaired two-tailed Student’s *t* test. *n* = 5 mice/genotype. Scale bar = 100 μm. (PDF 145 kb)

## Abbreviations

AMPK: Adenosine mono-phosphate (AMP)-regulated protein kinase
ANOVA: Analysis of variance
bFGF: Basic fibroblast growth factor
CIS: Cytokine-induced STAT inhibitor
CSA: Cross-sectional area
ES: Embryonic stem cell
G-CSF: Granulocyte colony stimulating factor
GH: Growth hormone
HRP: Horseradish peroxidase
Hz: Hertz
IFN-γ: Interferon-γ
IGF: Insulin-like growth factor
IL-6: Interleukin-6
JAK: Janus kinase
LPS: Lipopolysaccharide
MCK: Muscle creatine kinase
MKO: Muscle-specific knockout
MLC: Myosin light chain
MyHC: Myosin heavy chain
OCT: Optimal cutting temperature
PCR: Polymerase chain reaction
SDH: Succinate dehydrogenase
SEM: Standard error of the mean
SOCS: Suppressor of cytokine signaling
STAT: Signal transducers and activators of transcription
TA: Tibialis anterior
TNF-α: Tumor necrosis factor-α
UN: Uninjured
