# Supplementary figures and images for: Muscle-specific deletion of SOCS3 increases the early inflammatory response but does not affect regeneration after myotoxic injury
Source: Skelet Muscle. 2016 Oct 24;6:36. doi: 10.1186/s13395-016-0108-4 (PMC5078888; doi:10.1186/s13395-016-0108-4)

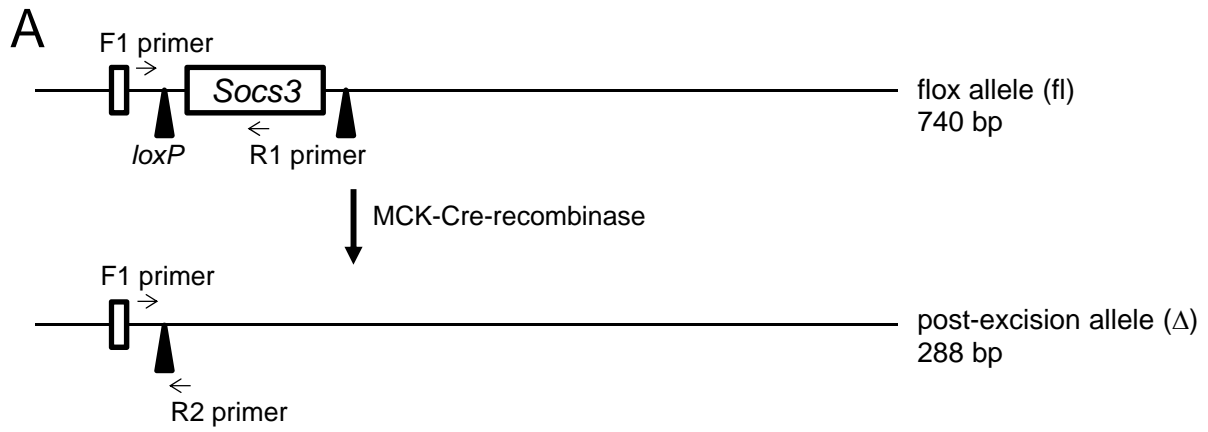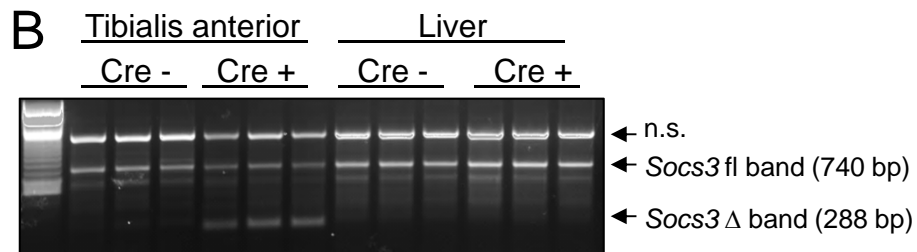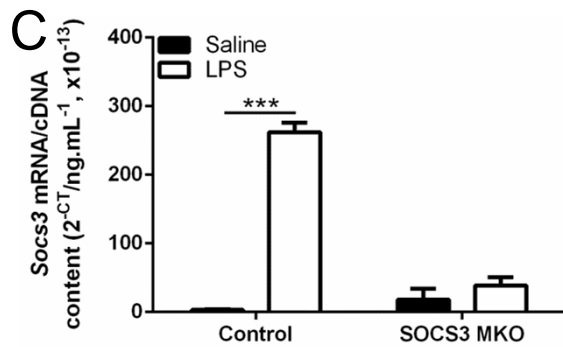

**Figure S1**

Supplement: Additional file 1: Figure S1. — Genomic PCR to confirm correct Socs3 deletion. (A) Schematic of Socs3 gene demonstrating floxed regions (LoxP) flanking exon 2 and location of the sequencing primers both prior to and post-Cre excision. (B) Genomic PCR using the primers shown in (A) confirmed the presence of the 288 bp deleted DNA band in muscle but not liver of SOCS3fl/fl MCK-Cre positive and not SOCS3fl/fl MCK-Cre-negative mice. (C) qRT-PCR using primers to detect Socs3 gene expression in RNA extracted from muscle fibers isolated from freeze-dried gastrocnemius muscles of saline or LPS-injected control and SOCS3 MKO mice. Data are expressed as mean ± SEM and compared with a two-way ANOVA and Fisher’s LSD post hoc multiple comparisons test to determine the effect of genotype and LPS injection (n = 2 mice/genotype). ***P < 0.001 compared to uninjured muscle from control mice. (PDF 115 kb) [file 13395_2016_108_MOESM1_ESM.pdf]

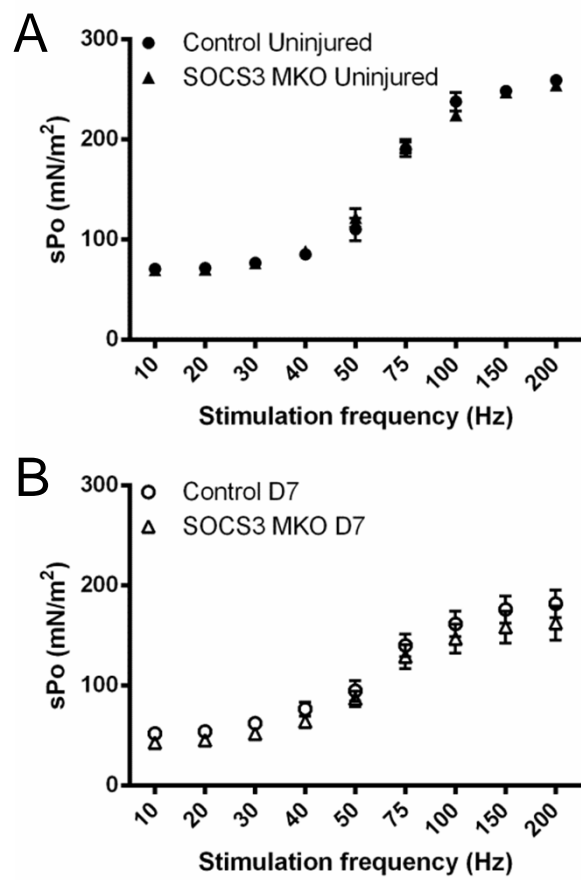

**Figure S2**

Supplement: Additional file 2: Figure S2. — Frequency-force relationships for uninjured and day 7 (D7) notexin-injured muscles from control and SOCS3 MKO mice. Frequency-force relations for specific (normalized) force at different stimulation frequencies (10–200 Hz) in muscles of either uninjured (A) or day 7 post-notexin-injured (B) control or SOCS3 MKO mice. Data are expressed as mean ± SEM. Comparisons were made using a two-way repeated measures ANOVA with Fisher’s LSD multiple comparisons test post hoc to determine the effect of genotype and stimulation frequency. n = 8 mice/genotype. (PDF 99 kb) [file 13395_2016_108_MOESM2_ESM.pdf]

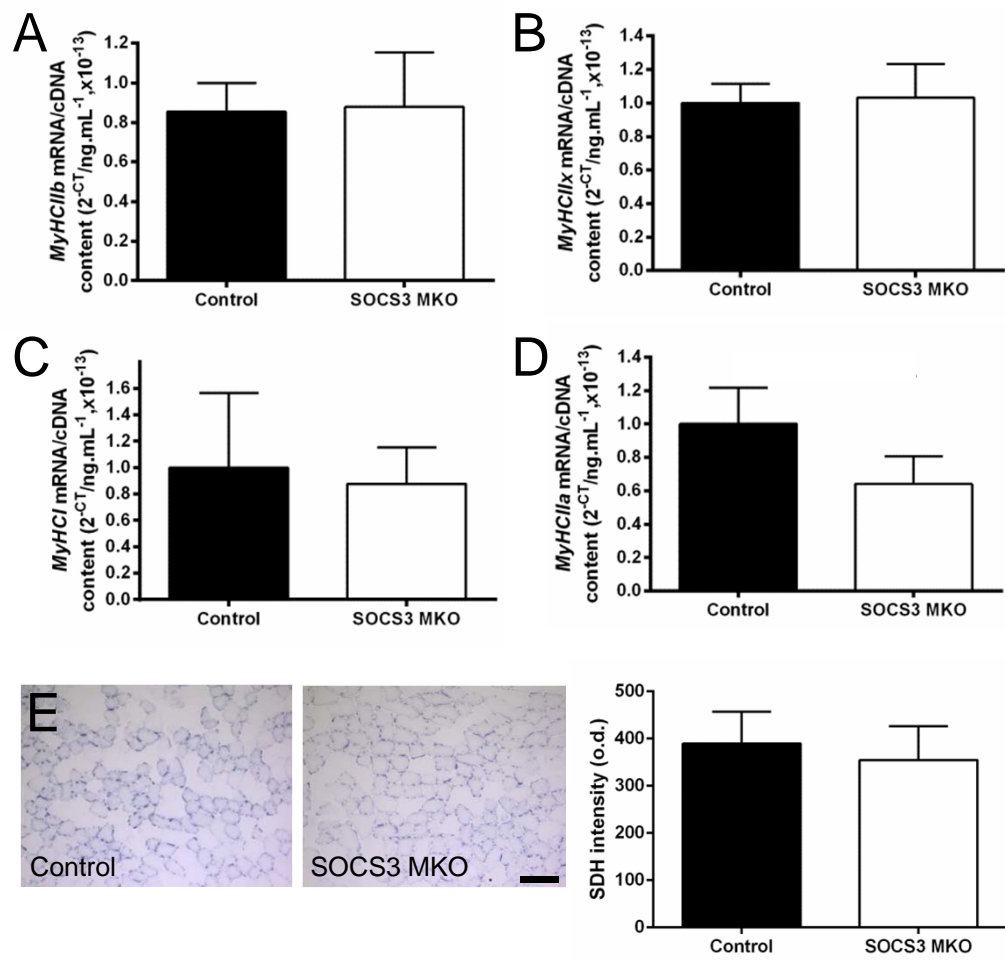

**Figure S3**

Supplement: Additional file 3: Figure S3. — Myosin heavy chain gene expression and muscle fiber oxidative capacity in muscles from uninjured control and SOCS3 MKO mice. qRT-PCR using primers to detect MyHCIIb (A), MyHCIIx (B), MyHCI (C), and MyHCIIa (D) was performed on RNA extracted from snap frozen muscles following dissection. Data are expressed as mean ± SEM and compared with an unpaired two-tailed Student’s t test. n = 8 mice/genotype. (E) Representative succinate dehydrogenase (SDH)-reacted TA muscle sections from uninjured muscles of 12-week-old control and SOCS3 MKO mice. Quantification of SDH intensity was determined by analysis of SDH reacted TA muscle sections. Data are expressed as mean ± SEM and compared with an unpaired two-tailed Student’s t test. n = 5 mice/genotype. Scale bar = 100 μm. (PDF 145 kb) [file 13395_2016_108_MOESM3_ESM.pdf]
